# Screen time and low back pain in children and adolescents: a systematic review of Brazilian studies

**DOI:** 10.1590/1984-0462/2023/41/2021342

**Published:** 2023-04-07

**Authors:** Paulo Henrique Guerra, Raquel Martelo, Maieli Naiara da Silva, Giovana Frazon de Andrade, Diego Giulliano Destro Christofaro, Mathias Roberto Loch

**Affiliations:** a Universidade Federal da Fronteira Sul, Chapecó, SC, Brazil.; b Universidade Estadual do Centro-Oeste, Guarapuava, PR, Brazil.; c Universidade Estadual Paulista, Presidente Prudente, SP, Brazil; d Universidade Estadual de Londrina, Londrina, PR, Brazil.

**Keywords:** Screen time, Low back pain, Child, Adolescent, Brazil, Review, Tempo de tela, Dor lombar, Crianças, Adolescente, Brasil, Revisão

## Abstract

**Objective:**

To identify and summarize the possible associations between screen time and low back pain in children and adolescents.

**Data source:**

Systematic searches were performed in five electronic databases (Lilacs, Scielo, Scopus, PubMed and Web of Science) on 01/25/2021, complemented by manual searches in reference lists and on Google Scholar, looking for original scientific articles that included Brazilian observational studies; whose samples had children and/or adolescents aged between 6 and 19 years, without specific clinical conditions, and that presented analyses of associations between indicators of screen time and nonspecific low back pain, based on regression models.

**Data synthesis:**

Nine cross-sectional studies whose samples had adolescents were included. Of the 18 analyses identified, nine reported risk relationships between the variables of interest. More specifically, risk associations were found in two studies that evaluated adolescents exposed to at least three hours using cell phone or tablet, and watching television per day. Also, instruments, cut-off points adopted, and screen equipment evaluated were diverse.

**Conclusions:**

Even though most of the risk associations were borderline from the statistical point of view, we found a higher frequency of risk associations between screen time and non-specific low back pain in adolescents exposed to screen time for at least three hours a day. In addition, further longitudinal studies with samples composed of children should be conducted across the country.

## Resumo

**Objetivo:**

Identificar e sumarizar os dados sobre as possíveis associações entre o tempo de tela e a dor lombar em crianças e adolescentes.

**Fontes de dados:**

Buscas sistemáticas em cinco bases de dados eletrônicas (Lilacs, Scielo, Scopus, Pubmed e Web of Science), no dia 25/01/2021, complementadas por buscas manuais em listas de referências e no site Google Acadêmico, procurando por artigos científicos originais que comunicaram estudos observacionais brasileiros que envolveram amostras de crianças e/ou adolescentes entre 6 e 19 anos de idade, sem condições clínicas específicas e que apresentassem análises de associações entre indicadores do tempo de tela e dor lombar não específica, baseadas em modelos de regressão.

**Síntese dos dados:**

Nove estudos transversais foram incluídos, envolvendo amostras de adolescentes. Das 18 análises identificadas, 9 mostraram relações de risco entre as variáveis de interesse. Mais especificamente, verificaram-se associações de risco em dois estudos que avaliaram adolescentes expostos a, pelo menos, três horas diárias de celular, tablete e televisão. Também se observou diversidade entre instrumentos, pontos de corte adotados e equipamentos de tela avaliados.

**Conclusões:**

Mesmo que a maior parte das associações de risco tenha sido limítrofe, do ponto de vista estatístico, a presente síntese apresentou maior frequência de associações de risco entre o tempo de tela e a dor lombar não específica em adolescentes expostos ao tempo de tela por, pelo menos, três horas diárias. Complementarmente, recomenda-se que sejam conduzidos no país estudos longitudinais, com o envolvimento de crianças nas amostras.

## INTRODUCTION

Sedentary behavior refers to activities that demand low energy expenditure while the person is awake and sitting, lying or reclining.^1^ Among different types of sedentary behavior, screen time can be highlighted, which represents individual or combined exposure to technological equipment with screens (e.g., cell phones, computers, tablets, televisions and video games). Screen time is one of the most frequently measured sedentary behaviors in studies involving children and adolescents.^2^

The specific concern of screen time is supported by the literature, given the associations between prolonged screen time and various negative health indicators in children and adolescents,^3^ such as non-specific low back pain,^4,5^ which is characterized as a multifactorial musculoskeletal discomfort and the main cause of disability in adulthood.^6^ Even though there is no consensus on the mechanisms of this relationship, one of the hypotheses is that low back pain is a consequence of excessive time spent in inadequate postures when using screen equipment.^7^ Since low back pain tends to increase throughout the life cycle,^8^ previous strategies related to its recognized risk factors are necessary for its control.

Similar to what international studies have reported, Brazilian studies suggest a high prevalence of screen time among both children and adolescents,^9^ considering the cutoff point of two hours a day recommended by international guidelines^10,11^ and adopted by a good portion of national research that addresses the theme,^2^ as well as low back pain.^12^ In order to support decision-making and guide steps for future national studies, the development of a synthesis based on national studies, which can indicate whether there are associations between these variables and verify the methodology adopted by the available studies. Thus, this paper aimed to identify and summarize data on possible associations between screen time and low back pain in children and adolescents, considering association studies developed in Brazil.

## METHOD

Given the greater concern to present the effects and magnitudes of possible associations between screen time and low back pain, with assessment of risk of bias in the studies, this paper proposes a systematic review of the literature previously recorded in the Prospero database (CRD42015025302). Its text is based on the items in the Preferred Reporting Items for Systematic Reviews and Meta-Analyses (PRISMA) checklist.^13^

The research question was defined per the acronym “PICOS”, with inclusion criteria for the synthesis of original scientific articles being:

1. Report of Brazilian observational studies;
2. Samples composed of children and/or adolescents aged between 6 and 19 years of,14 without specific clinical conditions (e.g., samples composed exclusively of children and/or adolescents with overweight, diabetes, some spinal anomaly, etc.) and;
3. Studies reporting associations between screen time indicators—investigated as an independent variable—and non-specific low back pain—investigated as a dependent variable—based on regression models.

The non-inclusion of studies involving children in early childhood (e.g., 0-5 years of age) is justified by the low prevalence of low back pain at this moment of life.^15^

The systematic searches were performed by three researchers in five electronic databases (Lilacs, Scielo, Scopus, PubMed and Web of Science), on January 25, 2021, based on the strategy developed for PubMed: (((((((low back pain[Text Word]) OR backache[Text Word]) OR sciatica[Text Word]) OR lumbago[Text Word])))) AND brazil[Text Word]. For Lilacs and Scielo databases, the searches also included Portuguese, using the terms: ((dor lombar) OR (lombalgia)) AND (Brasil). Complementary manual searches were carried out in the reference lists of full texts evaluated and by reading the first 200 records of the Google Scholar website (inserting terms, in English and Portuguese: “low back pain”, “sedentary”, “sitting time”, “screen time” and “Brazil”). There were no restrictions regarding year of publication. Articles written in Spanish, Portuguese or English were considered for the synthesis.

The evaluation by titles and abstracts was made by two independent researchers previously trained in systematic reviews, with the help of a third researcher to establish consensus. Data was collected from the original studies also by the two researchers, independently, with information divided into three domains:

1. Descriptive data of original articles (e.g., research location, year of data collection, sample size, percentage of female subjects in the sample, and age group);
2. Methodological aspects of articles (e.g., sample characteristics, screen time domain, evaluation method, screen time cut-off point, evaluation method, and prevalence of low back pain);
3. Method used to analyze association, measures of effect adopted, and results.

With the refinement of data from the collection worksheet, a descriptive synthesis was elaborated, following an organization by domains. As for the analyzes, in particular, only data obtained through regression analysis were considered for this review, regardless of the type of regression used (e.g., linear, logistic, Poisson). Recognizing that some articles could have different analysis types between screen time and low back pain, for example, stratifying by age group, sex or even specific types of screen equipment, it was previously stipulated that all data would be involved in the descriptive synthesis, which would be formulated based on the cut-off points and screen equipment analyzed aiming at a better understanding. In the case of studies that conducted crude and adjusted analyses, the data from the adjusted analyses were considered without losing sight of the strategy of controlling confounding factors. Finally, taking into account the estimates, magnitudes and p-value of the original data, screen time was classified as:

1. Risk factor;
2. Protection factor; or
3. Statistically null for low back pain.

The risk of bias of the articles selected was independently assessed by two researchers using a tool designed and developed based on items from internationally recommended tools: Effective Public Health Practice Project Tool (EPHPP),^16^ The Grading of Recommendations Assessment, Development and Evaluation (GRADE)^17^ and PRISMA.^14^ This tool is composed of 16 items organized into 3 domains:

1. Introduction (e.g., are the study objectives clearly presented?),
2. Methods (e.g., is the study design appropriate to meet its objective? Is the sample composed of a population free from specific clinical conditions? What is the justification for the sample size? What was the sampling process? What is the sample representativeness? Was there prior validation and information that allows the replication of instruments used to measure screen time and low back pain, and the adequacy of the analysis protocol? Was there control of confounding factors?);
3. Results (e.g., are data adequately described and have internal consistency?).

The instrument can be requested with the corresponding author, via email.

## RESULTS

**Figure 1.**
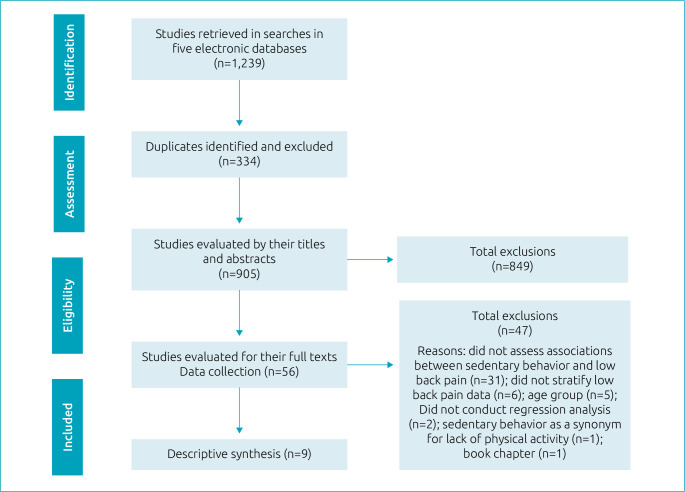
Flowchart of the systematic review.

After applying the search strategies in the five electronic databases, 1,239 references were retrieved, 334 of which were identified as duplicates and subsequently excluded. Thus, 905 references were evaluated per their titles and abstracts. Of these, 56 were kept for full text evaluation. At the end of this stage, with the exclusion of 47 references, whose main reasons were “absence of analysis of association between screen time and low back pain” (n=31) and “studies that evaluated the spine, but without stratification of lumbar region” (n=6), 9 original articles,^18-26^ with a cross-sectional design were picked for the synthesis (Figure 1).

The surveys had been carried out in nine cities of five Brazilian states (Table 1). More specifically, five surveys (55.6%) had been carried out in municipalities of the state of São Paulo (Bauru,^18,19^ Itaquaquecetuba and Mogi das Cruzes,^21^ Ourinhos^20^ and São Paulo^26^). Altogether, data collection took place between 2007^19^ and 2017,^18,21^ with the identification of different procedures for sample composition, such as including all adolescents in the city of Caracol (Piauí),^22^ all students enrolled in municipal public schools,^19,20^ in addition to samples composed of probabilistic and non-probabilistic processes (Table 1). In terms of size, the samples varied between 330^24^ and 1,628 subjects,^18^ most of them being female participants in six of the eight studies that made this information available (75%).^19,22-26^ Regarding age groups, all studies involved adolescents (≥11 years of age).^18-26^

**Table 1.** Descriptive information of included studies (n = 9).

Reference	Research location (year of data collection)	Sampling	Sample (% of females)	Age group
Bento et al.^18^	Bauru (SP); 2017	R	1,628 (nd)	14–18
De Vitta et al.^19^	Bauru (SP); 2007	AE	1,236 (52)	11–14
Fernandes et al.^20^	Ourinhos (SP); 2009	AE	1,461 (48)	10–14
França et al.^21^	Itaquaquecetuba and Mogi das Cruzes (SP); 2017	C	577 (48)	10–16
Meucci et al.^22^	Caracol (PI); 2010	AT	1,112 (53)	13–19
Onofrio et al.^23^	Pelotas (RS); 2009	R	1,280 (54)	13–19
Schwertner et al.^24^	Florianópolis (SC); NR	C	330 (74)	15–18
Silva et al.^25^	Recife (PE); 2012	R	961 (59)	14–19
Zapata et al.^26^	São Paulo (SP); NR	C	791 (52)	14–17

R: sampling by randomized process; AE: all enrolled in municipal public schools; C: convenience sample; AT: all teenagers in the city; NR: not reported; nd: not described.

In the risk of bias assessment (Table 2), weaknesses were related to the absence of reports on the representativeness of the sample (n=6; 66.7%), on prior screen-time measurement instrument validation (n=8; 88.9%), and on the blinding of statistical analysis, in relation to exposures and outcomes (n=9; 100%). On the other hand, adequacy of the design in relation to the objectives of the studies, presentation of information that allow the replication of measures used to assess low back pain, statistical analysis and strategies to control confounding factors were assessed in all studies (n=9; 100%).

**Table 2.** Assessment of risk of bias in included studies (n=9).

Assessed items	Yes	No	Not reported
1. Presentation of the study objectives.	9^18-26^	0	0
2. Robustness of the design, considering the objective of the study.	9^18-26^	0	0
3. Is the sample heterogeneous?	8^18-23,25,26^	0	1^24^
4. Was the sample drawn from an adequate population base representing the target population under investigation?	7^18-23,25^	2^24,26^	0
5. Sample size justification.	7^18-20,22,23,25,26^	2^21,24^	0
6. Report of sample representativeness/Is the sample representative?	3^22,23,25^	6^18-21,24,26^	0
7. Is there a presentation of the number of non-respondents, with justifications?	6^19,20,22,23,25,26^	3^18,21,24^	0
8. Is there a report of previous validation of the instrument used to measure screen time?	1^23^	8^18-22,24-26^	0
9. Is there information that makes it possible to replicate the instrument used to measure low back pain?	6^18-20,22,23,26^	3^21,24,25^	0
10. Is there a report of previous validation of the instrument used to measure low back pain?	7^18-22,24,25^	2^23,26^	0
11. Is there information that makes it possible to replicate the instrument used to measure low back pain?	9^18-26^	0	0
12. Are the procedures used in the statistical analysis adequate?	9^18-26^	0	0
13. Is there an indication of who conducted the statistical analysis?	0	9^18-26^	0
14. Is there a strategy to control the most relevant confounding factors?	9^18-26^	0	0
15. Are data adequately described?	9^18-26^	0	0
16. Are results internally consistent?	9^18-26^	0	0

Seven studies measured screen time using their own questionnaires (77.8%),^18,21-26^ measuring different screen time indicators and the cutoff point of two hours per day (n=7; 77.8%) (Table 3). Regarding questionnaires to measure non-specific low back pain, the Nordic Questionnaire was more often used (n=4),^18-20,22^ as it investigates symptoms of low back pain in the last 12 months. Regarding procedures used in the analyses, logistic regressions were more often used to assess possible associations between screen time and low back pain (n=6; 66.7%).^19-21,24,26^

**Table 3.** Description of instruments and cut-off points used to assess sedentary behavior and low back pain (n=9).

References	Screen time assessment tool (cut-off points)	Low back pain assessment tool (prevalence period)	Type of regression
Bento et al.^18^	OQ (3h/d)	Nordic Questionnaire (last 12 months)	Poisson
De Vitta et al.^19^	Questionnaire by Harreby et al. (2 h/d)	Adapted Nordic Questionnaire (last 12 months)	Logistic
Fernandes et al.^20^	Questionnaire by Harreby et al. (2 h/d)	Adapted Nordic Questionnaire (last 12 months)	Logistic
França et al.^21^	OQ (2 h/d)	Back Pain Assessment Instrument (moment of interview)	Logistic
Meucci et al.^22^	OQ (2 h/d)	Adapted Nordic Questionnaire (last 12 months)	Poisson
Onofrio et al.^23^	OQ (2 h/d)	OQ (last month)	Poisson
Schwertner et al.^24^	OQ (2 h/d)	Oliveira - Questionnaire on Low Back Pain in Youths (moment of interview)	Logistic
Silva et al.^25^	OQ (4 h/d)	OQ (last 6 months)	Logistic
Zapata et al.^26^	OQ (2 h)	OQ and physical evaluation	Logistic

OQ: own questionnaire; h: hours; h/d: hours per day.

Table 4 shows a summary of the 18 analyses on screen time versus low back pain conducted with adolescents, in which 9 suggest risk associations between the variables (50%). Based on stratification by exposure time and types of screen, a higher frequency of risk associations was observed in the stratum exposed to at least three hours of cell phone use (prevalence ratio – adjusted PR=1.4; confidence interval – 95%CI 1.1–1.7),^18^ tablet (adjusted PR=1.5; 95%CI 1.2–1.8)^18^, and television (adjusted PR=1.2; 95%CI 1.0–1.4^18^ and Odds Ratio – adjusted OR=1.5; 95%CI 1.0–2.3).^20^

**Table 4.** Synthesis of results, stratified by cut-off points and screen equipment evaluated (n=9).

Cut-off points	Type of screen and results
≥2 hours per week	Computer: week days: RR=1.5 (95%CI 1.0–2.1)^26^
	Computer: weekends: RR=1.8 (95%CI 1.4–2.4)^26^
≥2 hours per day	Computer: Non-adjusted RP=1.2 (95%CI 0.9–1.8)^23^ Computer and television: Adjusted OR=0.3 (95%CI 0.1–1.4)^* 24^; Adjusted OR=3.0 (95%CI 0.7–13.9)^† 24^ Television: Adjusted OR=1.9 (95%CI 1.3–2.7)^19^; Non-adjusted RP=1.0 (95%CI 0.8–1.2)^22^; Non-adjusted RP=0.9 (95%CI 0.7–1.2)^23^ Cell phone, computer, tablet, television and video games: no risk associations^21^ Use of more than one screen device: girls: no risk associations^21^; boys: Adjusted OR=0.3 (95%CI 0.1–0.9)^21^
≥3 hours per day	Cell phone: Adjusted RP=1.4 (95%CI 1.1–1.7)^18^ Tablet: Adjusted RP=1.5 (95%CI 1.2–1.8)^18^ Television: Adjusted RP=1.2 (95%CI 1.0–1.4)18; Adjusted OR=1.5 (95%CI 1.0–2.3)^‡ 20^
≥4 hours per day	Computer/games: Adjusted OR=1.3 (95%CI 1.0–1.8)^25^
≥5 hours per day	Computer: Non-adjusted RP=1.2 (95%CI 0.8–1.8)^23^ Television: Non-adjusted RP=0.7 (95%CI 0.5–1.2)^23^

* low back pain at the time of study;

† low back pain at some point in life;

‡ >3 times a week and 3 hours a day; 95%CI: 95% confidence interval; OR: Odds Ratio (odds ratio); PR: prevalence ratio; RR: relative risk.

## DISCUSSION

Altogether, the synthesis of this review consisted of nine original Brazilian studies, who conducted 18 analyses investigating possible associations between screen time and low back pain.^18-26^ Of these, nine showed risk relationships between screen time indicators and non-specific low back pain.^18-21,25,26^ When stratified by cut-off points, the synthesis indicated risk associations in two studies that evaluated adolescents exposed at least three hours a day to cell phones,^18^ tablets^18^ and television.^18,20^ In relation to the internationally recommended cut-off point^10,11^ of two hours a day for screen time, only two of the studies pointed to risk associations in exposure to television^19^ and combined time on different screen devices among boys.^21^

Overall, this result corroborates previous reviews^4,5^ that also involved international studies in their syntheses. However, when comparing our results with those of the review by Silva et al.,^5^ it is worth mentioning that our synthesis indicates a higher frequency of risk associations with low back pain by the cutoff point of at least three hours a day in different screen equipment. As mentioned in the introduction, it is possible that low back pain is triggered by prolonged inadequate postures while using screen equipment.

The use of different tools to measure the variables of interest, such as the technological dynamics of screen equipment, can largely justify the oscillating, borderline results or even the lack of association between variables in the original studies. Regarding the instruments, in most of the included studies, there was heterogeneity between questionnaires used to measure screen time, screen equipment assessed and cut-off points to determine high exposure, like the identification of different periods of prevalence of low back pain.

This assortment limited further comparisons between findings. In this sense, future studies are suggested to use, in parallel with motion sensors (which allow objective measurements), previously validated questionnaires to measure screen time, guaranteeing robustness to the exposure time and allowing a better understanding of the contexts and screen equipment used. Looking at the national production on sedentary behavior, there is a lack of data on previous validations of questionnaires used.^2^ This is an important limitation, as studies cannot be compared and replicated.

In terms of practical application, the understanding that recreational screen time is not just an individual option for children and adolescents can be reinforced. In Brazil, for example, having a higher income and living in urban spaces are factors associated with sedentary behavior.^27^ Thus, strategies aimed at reducing or controlling it must be carefully designed and have a broader focus, supported by the ideals of promoting health at all stages of actions. Two not-competing alternatives are suggested in the following paragraphs.

From the perspective of information, one can point out the potential of educational approaches to reduce screen time,^28^ based on less specific and prescriptive messages, so that children and/or adolescents (or their parents, caregivers and teachers) can adapt them to their possibilities. The Physical Activity Guide for the Brazilian population,^1^ for example, brings messages that can be adopted in different contexts, such as: “the shorter the time spent in sedentary behavior, the better” and “whenever possible, reduce the time you spend sitting or lying down watching television or using your cell phone, computer, tablet or video game”.^1^ It is understood that the data from this synthesis can also be used to support strategies that seek to inform risks and reduce sedentary behaviors in Brazilian adolescents.

From the perspective of valuing what “competes” with screen equipment in time management, when one understands that the options offered by screen equipment are quite attractive not just for children and adolescents, it is important to recognize and value behaviors that “compete” directly with the time spent on screen equipment. Given the inverse associations between screen time and physical activity,^29,30^ one of the possible ways to reverse the high exposure to screen equipment is creating/revitalizing different leisure options that allow the practice of physical activity. In this sense, it is worth mentioning that these spaces should be accessible, easy, convenient and valued among children and adolescents, considering their perceptions, interests and possibilities.

Apart from the methodological procedures adopted, some limitations should be mentioned: in view of the small number of studies and the borderline associations between the cutoff of three hours of screen time per day and low back pain, caution is recommended in extrapolating this result; even if “low back pain” was defined in the eligibility criteria as a dependent variable, since the synthesis was composed only of cross-sectional studies, there may be the possibility of reverse causality (e.g., children/adolescents spend more time sitting, exposed to screens, as they feel low back pain). So, one can also suggest longitudinal studies in the country to follow up children from school initiation to the end of adolescence, aiming at a more robust measure on the possible relation between screen time and low back pain.

In conclusion, even though most risk associations were borderline from a statistical point of view, this review showed a higher frequency of risk associations between screen time and non-specific low back pain in adolescents exposed to screens for at least three hours a day. In addition, it is recommended that longitudinal studies with samples involving children be conducted in the country.
